# Patient preferences for the diagnosis of coeliac disease: A discrete choice experiment

**DOI:** 10.1002/ueg2.12651

**Published:** 2024-08-27

**Authors:** Mohamed G. Shiha, Nyantara Wickramasekera, Suneil A. Raju, Hugo A. Penny, David S. Sanders

**Affiliations:** ^1^ Division of Clinical Medicine School of Medicine and Population Health University of Sheffield Sheffield UK; ^2^ Academic Unit of Gastroenterology Sheffield Teaching Hospitals Sheffield UK; ^3^ Sheffield Centre for Health and Related Research University of Sheffield Sheffield UK

**Keywords:** accuracy, adult, antibodies, biopsy, coeliac disease, discrete choice experiment, endoscopy, preference, serology, survey

## Abstract

**Background:**

There is potential for a paradigm shift from a biopsy‐to a serology‐based diagnosis of coeliac disease in selected adult patients. However, it remains unknown if this approach would be acceptable to patients. We aimed to explore patients' preferences regarding the no‐biopsy approach for coeliac disease diagnosis.

**Methods:**

We developed a discrete choice experiment survey containing 12 different scenarios with two possible alternatives (endoscopy & biopsy or serology) to estimate patient preferences. The scenarios were based on 5 attributes: risk of false positive results, risk of missed diagnosis, waiting time to start treatment, risk of complications, discomfort, or pain. Patient preferences and the relative importance of the attributes were estimated using a mixed logit model.

**Results:**

In total, 385 people (70.6% female, 98.2% white) across the four nations of the United Kingdom completed the survey. Respondents preferred a serology‐based diagnosis over endoscopy and duodenal biopsies (59% vs. 41%, β coefficient 1.54, *p* < 0.001). Diagnostic test accuracy (*p* < 0.001), shorter waiting time to start treatment (*p* < 0.001), and discomfort levels during the procedure (*p* < 0.001) were the most important attributes to respondents. The risk of complications, including perforation and bleeding, did not significantly influence respondents' choices. Respondents with previous endoscopy experience were more willing to undergo endoscopy compared with those who never had one.

**Conclusion:**

The no‐biopsy approach to diagnosing coeliac disease is acceptable and preferred by patients over endoscopy and biopsy. Our findings highlight the importance of patient‐centred care and shared decision‐making in guiding diagnostic strategies for optimal patient outcomes.

## Graphical abstract

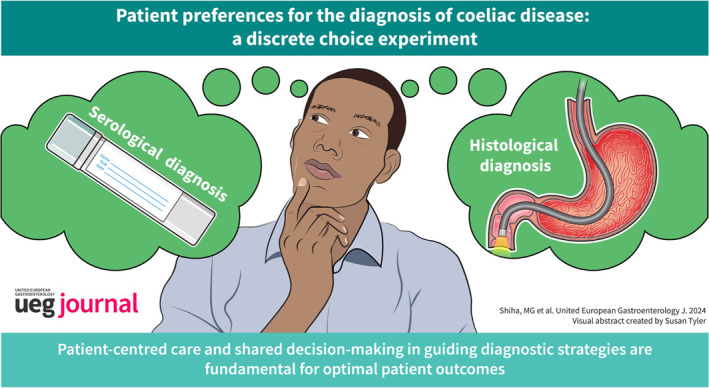

**Key summary**

**Summarise the established knowledge on this subject**

- Coeliac disease has traditionally been diagnosed through endoscopy and biopsy, but new evidence suggests that serology alone may be sufficient for diagnosis in selected adult patients.
- Despite over a decade of ongoing debate among gastroenterologists regarding adopting a no‐biopsy approach to diagnosing coeliac disease in adults, patient preferences have been largely overlooked.

**What are the significant and/or new findings of this study?**

- Using a discrete choice experiment, we found that patients prefer serology‐based diagnosis of coeliac disease over endoscopy and biopsy.
- Diagnostic test accuracy, waiting time to start treatment, and procedural discomfort are critical factors influencing patient preferences.

## INTRODUCTION

Coeliac disease is a chronic autoimmune disorder triggered by dietary gluten in genetically predisposed individuals. It is characterised by immune‐mediated inflammation in the small intestinal mucosa, which leads to villous atrophy and a wide range of gastrointestinal and extra‐internal symptoms.^1^ The prevalence and incidence of coeliac disease has been increasing worldwide, with current estimates suggesting that approximately 1% of the global population is affected.^2^, ^3^ However, many patients with coeliac disease remain undiagnosed or experience significant delays in diagnosis.^4^

The current diagnostic pathway for coeliac disease is a two‐step process: initial testing for coeliac‐specific serological antibodies, such as anti‐tissue transglutaminase (tTG) and endomysial antibodies (EMA), followed by endoscopy and confirmatory biopsy in serology‐positive cases.^5^ However, the need for intestinal biopsy can be a barrier to diagnosis due to its invasive nature and the associated discomfort and risks of endoscopy. This requirement may deter some patients from completing the diagnostic evaluation, potentially leaving their coeliac disease undiagnosed and untreated. Therefore, the European paediatric guidelines adopted a no‐biopsy approach to diagnosing coeliac disease in children with very high IgA‐TTG titres (>10 times the upper limit of normal) and a positive EMA in a second blood sample.^6^ This no‐biopsy approach has been shown to be highly accurate in children, with a positive predictive value (PPV) of >99%.^7^

Evidence supporting the high accuracy of the no‐biopsy approach has been extrapolated to the adult population. Several studies found that IgA‐TTG ≥10 × ULN could be diagnosed without biopsy, with PPV ranging between 95% and 99%.^8^, ^9^, ^10^ However, adopting this strategy in adults remains contentious.^11^ The latest American College of Gastroenterology Guidelines did not broadly adopt the no‐biopsy approach, citing concerns that the PPV of 95% may be unacceptably low given the implications of a lifelong gluten‐free diet.^12^

In this ongoing debate over whether to adopt the no‐biopsy approach for diagnosing coeliac disease in adults, a critical aspect often overlooked is the inclusion of patient values and preferences. A discrete choice experiment (DCE) is a powerful quantitative method used to elicit individual preferences, and the trade‐offs patients are willing to make when choosing between different attributes of healthcare interventions.^13^ In a DCE, patients are presented with a series of hypothetical scenarios, each consisting of different combinations of attributes, and are asked to choose their preferred option in each scenario. This method has been shown to accurately predict the choices that individuals are likely to make in real‐world situations.^14^ In this study, we used a DCE to explore patient preferences for the diagnostic methods of coeliac disease and to assess the acceptability of a serology‐based diagnosis of coeliac disease in adults.

## METHODS

### Qualitative phase and selection of attributes

To ensure relevant attribute selection for our DCE, we performed a comprehensive literature review to identify potential attributes that may influence patients' preferences when choosing a diagnostic approach for coeliac disease. This was followed by semi‐structured interviews with a patient advisory group consisting of 8 patients with coeliac disease (median age 48.5 years, 75% female). The interviews focussed on the participants' diagnostic journeys, exploring the positive and challenging aspects of their experiences with coeliac disease. We asked guiding questions while providing space for participants to express their perspectives. Participants were then asked to rank various attributes based on what they perceived as the most important.^15^ Based on this qualitative phase of the study, the research team selected five key attributes to be included in the DCE survey (Table 1). The levels of all attributes were based on the published literature and real‐world practice to ensure their relevance and validity.^10^, ^16^, ^17^, ^18^

**TABLE 1:** Attributes and levels included in the DCE model.

Attribute	Endoscopy with biopsy	Serology
Risk of wrong diagnosis	None	2 out of every 100 people (2%)
		5 out of every 100 people (5%)
		35 out of every 100 people (35%)
Risk of missed diagnosis	None	None
	2 out of every 100 people (2%)	
	10 out of every 100 people (10%)	
Waiting time to start treatment	2 months	2 weeks
	3 months	1 month
	6 months	2 months
Risk of complications	Bleeding (0.1%)	None
	Perforation (0.02%)	
Discomfort/pain	Minimal	Minimal
	Moderate	Moderate
	High	

Abbreviation: DCE, discrete choice experiment.

### DCE design and pilot study

**FIGURE 1:**
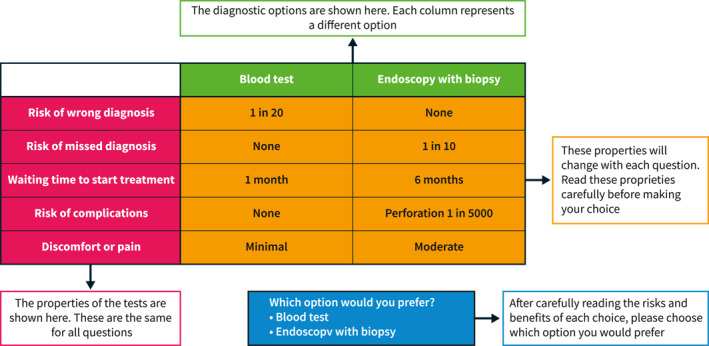
Sample question with an example of choice set.

We used the selected attributes and levels to generate a D‐efficient experimental design with level balance. A labelled design was generated using NGene software (ChoiceMetrics). The online survey was created using the Qualtrics platform and consisted of the DCE followed by demographic questions to capture participants' characteristics. The survey introduction included information about coeliac disease diagnosis and an explanation of all the included attributes. A sample DCE scenario (Figure 1) was shown to patients before starting the survey to familiarise them with the structure and format of the questionnaire. Each participant was randomly assigned to one of two blocks of 12 hypothetical choice sets, each containing different combinations of attribute levels and two diagnostic options (endoscopy with biopsy and serology).

We conducted a pilot study with the patient advisory group to ensure that the survey was clear and comprehensible to all patients with coeliac disease. Feedback from this pilot study was incorporated to improve the survey design. The survey, including four representative choice sets, is available in the supplementary material.

No standard exists for sample size in DCE studies.^19^ Therefore, we aimed to collect data from approximately 300 people based on the relevant published literature.^20^

### Study population and survey dissemination

We invited adults aged 18 and older with confirmed or suspected coeliac disease to participate in an online survey via email through Coeliac UK charity in February 2024. Participants were provided with a comprehensive information sheet describing the study, and those who agreed to participate provided consent and were directed to the online survey on the Qualtrics platform. Data were collected anonymously. No incentives were provided for participation in the study.

### Statistical analysis

Descriptive statistics were used to describe demographic data; continuous variables were summarised using medians and interquartile ranges, while categorical variables were presented as frequencies and percentages. Patients' preferences were analysed using a mixed multinomial logit model.^21^ The model was estimated with simulated maximum likelihood using 1000 Halton draws. Attributes were dummy coded and assumed to be normally distributed. Relative importance scores were calculated as the difference between the most and least preferred levels in each attribute, divided by the sum of the average differences for all attributes, scaled by 100. A latent class multinomial logit model was then used to identify distinct patient groups with similar diagnostic preferences. Following the identification of the optimal number of latent classes (using model fit statistics), we incorporated sociodemographic characteristics into the model to identify distinct patient groups with similar diagnostic preferences. We calculated trade‐offs (marginal rates of substitution [MRS]) using the results of the latent class model. The trade‐offs were calculated by dividing the coefficient of all attribute levels (e.g., waiting time) by the coefficient of the risk of a wrong diagnosis. Confidence intervals were calculated using the delta method. A *p*‐value of <0.05 was considered statistically significant. All statistical analyses were performed using Stata version 18 (StataCorp., College Station).

## RESULTS

### Participants characteristics

The survey was emailed to a database of 5000 members of Coeliac UK on the 28th of February, 2024 and was concluded on the 11th of March, 2024 after exceeding the target number of responses. A total of 562 responses were received for the survey. Of these, 385 people completed the survey in full and were included in the study. The median time to complete the survey was 7.3 min (IQR 5.4–10.0 min). The majority of respondents were females (70.6%), white (98.2%) and over 55 years old (68.8%). Most respondents had a confirmed diagnosis of coeliac disease (94.8%), and 85.7% had previous endoscopy experience. The baseline characteristics of the study participants are presented in Table 2.

**TABLE 2:** Participants characteristics.

	Participants (*n* = 385)
Sex	
Female	272 (70.6%)
Male	110 (28.6%)
Prefer not to say	3 (0.8%)
Age group	
<25	10 (2.6%)
25–34	31 (8.1%)
35–44	24 (6.2%)
45–55	55 (14.3%)
>55	265 (68.8%)
Ethnicity	
White	378 (98.2%)
Asian or Asian British	1 (0.3%)
Black, Black British, Caribbean or African	1 (0.3%)
Other ethnic background	4 (1.0%)
Prefer not to say	1 (0.3%)
Education	
University or postgraduate	196 (50.9%)
College or technical school	107 (27.8%)
High school or below	82 (21.3%)
Employment	
Employed, full‐time or part‐time	162 (42.1%)
Retired	203 (52.7%)
Student	9 (2.3%)
Unemployed	11 (2.9%)
Residence	
South of England	159 (41.3%)
North of England	94 (24.4%)
Midlands	71 (18.4%)
Scotland	33 (8.6%)
Wales	22 (5.7%)
Northern Ireland	6 (1.6%)
Coeliac disease diagnosis	
Confirmed	365 (94.8%)
Suspected	20 (5.2%)
Endoscopy experience	
Yes	330 (85.7)
No	55 (14.3)

### Patient preferences for diagnosis

**FIGURE 2:**
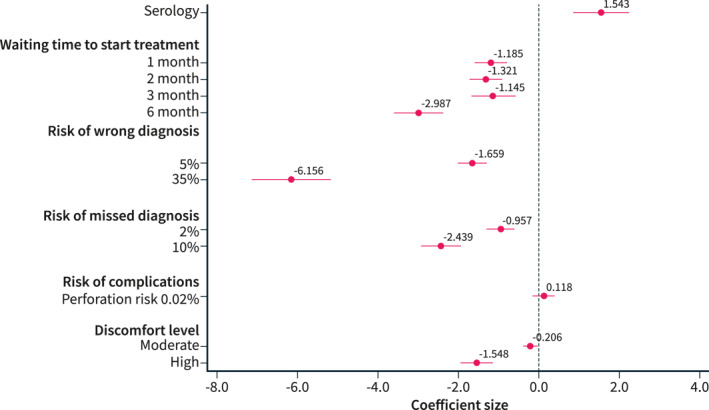
The results of the mixed logit model of patient preferences for the diagnosis of coeliac disease.

As shown in Figure 2 and Table S1, the results of the mixed logit model confirmed a strong preference for serology over endoscopy and biopsy (59% vs. 41%, β coefficient 1.54, *p* < 0.001). Longer waiting times to start treatment, particularly 6 months, were associated with reduced preferences (β coefficient −2.98, *p* < 0.001). The risk of wrong diagnosis at 35% (β coefficient −6.15, *p* < 0.001), the risk of missed diagnosis at 10% (β coefficient −2.43, *p* < 0.001), and high levels of discomfort (β coefficient −1.54, *p* < 0.001) were the most significant deterrents for patients when choosing a diagnostic approach. Conversely, the small risk of complications did not significantly influence patient preferences (β coefficient −1.11, *p* = 0.39).

**FIGURE 3:**
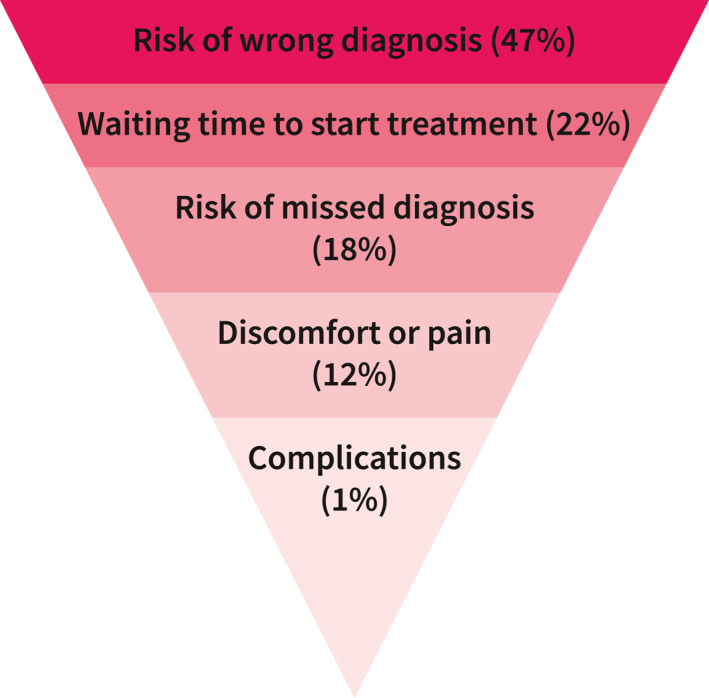
The relative importance of attributes to patients.

The relative importance of the diagnostic attributes is illustrated in Figure 3. The risk of wrong diagnosis (47%) was the diagnosis tant factor influencing patients' decisions, followed by waiting time to start treatment (22%), risk of missed diagnosis (18%), discomfort or pain during the procedure (12%), and risk of complications (1%).

### Latent class analysis

The latent class analysis (Table 3) identified 2 classes of patients with distinct preferences: patients who favoured serology (Class 1) and those who favoured endoscopy (Class 2). Similar to the results of the mixed logit model, the preferences of both classes were influenced by diagnostic accuracy, waiting time to start treatment, and risk of high discomfort. Female participants (β coefficient 0.64, *p* < 0.05) and those with no previous endoscopy experience (β coefficient 1.86, *p* < 0.001) were more likely to belong to Class 1, favouring serology over endoscopy. Age, education level, employment status and ethnicity did not significantly impact class membership. The trade‐offs based on the willingness to accept the risk of a wrong diagnosis for the different attribute levels based on the latent class model results are shown in Table S2. In Class 1, participants' willingness to accept the risk of a wrong diagnosis (MRS 1.49, 95% CI 1.31–1.67) suggests that they are willing to accept a slightly higher chance of a wrong diagnosis to avoid an endoscopy. Conversely, in Class 2, participants demonstrated a stronger aversion with a negative willingness to accept the risk of a wrong diagnosis (MRS −1.03, 95% CI −1.44 to −0.63). Across both classes, the negative willingness to accept risk for most of the attribute levels suggests an unwillingness to tolerate the less desirable aspects of diagnostic procedures (e.g., longer wait times or high discomfort) unless there was a corresponding improvement in diagnostic accuracy.

**TABLE 3:** Latent class analysis with class membership.

	Class 1	Class 2
	(Prefer serology)	(Prefer endoscopy)
	60.8%	39.2%
Coefficient (standard error)		
Serology	2.96*** (0.25)	−1.24*** (0.20)
Waiting time to start treatment		
1 month	−0.65** (0.20)	−0.70*** (0.17)
2 months	−0.66** (0.21)	−0.578*** (0.17)
3 months	−0.50 (0.26)	−0.15 (0.23)
6 months	−1.52*** (0.19)	−1.04*** (0.27)
Risk of wrong diagnosis	−1.986*** (0.11)	−1.20*** (0.08)
Risk of missed diagnosis	−0.62*** (0.09)	−0.66*** (0.08)
Perforation risk 0.02%	0.10 (0.11)	0.00 (0.13)
Discomfort level		
Moderate	−0.15* (0.070)	−0.08 (0.080)
High	−0.75*** (0.142)	−0.79*** (0.150)
Class membership		
Female (ref: Male)	0.64* (0.25)	Reference
Age (reference >55 years)		
<25	−0.75 (0.85)	
45–55	−0.31 (0.44)	
35–44	−0.42 (0.55)	
25–34	−0.11 (0.53)	
Education (reference: College/technical school)		
High school or below	0.42 (0.33)	
University or postgraduate	0.07 (0.28)	
No formal coeliac diagnosis	−1.15 (0.68)	
No endoscopy experience	1.86*** (0.53)	
Employment (reference: Employed)		
Retired	−0.23 (0.333)	
Student	−0.44 (0.91)	
Unemployed	−0.95 (0.71)	
Ethnicity (not white or not disclosed)		
White	1.74 (1.13)	

*p < 0.05, **p < 0.01, ***p < 0.001.

## DISCUSSION

To our knowledge, this is the first study to investigate patient preferences for the diagnostic methods of coeliac disease. We used a DCE design to quantify the trade‐offs that patients are willing to make when choosing between a serology‐ and biopsy‐based diagnosis. Our results showed that adult patients prefer the non‐invasive, no‐biopsy approach over endoscopy and biopsy. The trade‐offs between diagnostic accuracy, waiting time to start treatment and procedural discomfort significantly influenced patient preferences. Interestingly, male participants and those with previous endoscopy experience were more likely to prefer endoscopy and biopsy over serology.

The strong preference for serology over endoscopy highlights patients' desire for non‐invasive diagnostic tests that provide accurate and timely results. While diagnostic accuracy remains important, the perceived invasiveness of endoscopy, including potential discomfort and longer waiting times, likely deterred some patients from choosing it. This aligns with the established patient preference for less‐invasive approaches in other conditions. For example, studies on inflammatory bowel disease showed that patients prefer stool testing and intestinal ultrasound over endoscopy to monitor their disease activity.^22^, ^23^ In a recent meta‐analysis, we found that the PPV of the no‐biopsy approach to identify patients with coeliac disease was 65%, 88%, 95%, and 99% if the pre‐test probability of coeliac disease was 1%, 4%, 10% and 40%, respectively.^10^ It has been postulated that the PPV of 95% may be unacceptably low.^12^ However, our findings confirm that some patients may be willing to accept a slightly lower diagnostic accuracy if it means avoiding more invasive procedures such as endoscopy.

Longer waiting times, particularly the 6‐month delay to start treatment, were the most significant deterrent influencing patients' decision to opt for endoscopy and biopsy. The latest national data from the UK showed that only 18% of NHS services met the 6‐week waiting time target for routine endoscopy.^24^ Patients with suspected coeliac disease often face a mid‐diagnostic uncertainty between the time of their positive serological tests and the confirmation of their diagnosis with endoscopy and biopsy.^25^ During this time, which may extend to several months, patients are advised to continue eating gluten even if symptomatic to avoid false negative results during endoscopy. Those who continue to consume a gluten‐containing diet while awaiting endoscopy not only experience worsening symptoms but also significant distress as they are aware that their symptoms will likely improve once they are able to eliminate gluten from their diet.^25^ The negative effects of waiting for endoscopy are likely compounded by the diagnostic delays that many patients with coeliac disease experience, which may extend to several years.^26^, ^27^

Endoscopy is often perceived as an uncomfortable and daunting procedure. High levels of discomfort during the procedure were significantly associated with a decrease in patients' willingness to choose endoscopy over serology. Conversely, the rare risk of serious complications such as bleeding or perforation did not deter patients from choosing endoscopy. Patient anxiety about endoscopy occurs while waiting for the test, during the actual procedure, and while awaiting the results.^28^ We found that patients with previous endoscopy experience were more willing to choose endoscopy over serology than those who had never had one. Familiarity with the procedure and knowing what to expect may reduce anxiety for some patients.^28^, ^29^ Moreover, individuals with prior endoscopy experience may have developed coping mechanisms to deal with the discomfort and anxiety of endoscopy. Importantly, those with biopsy‐confirmed diagnoses may perceive endoscopy as the most definitive and gold‐standard diagnostic tool despite its invasive nature.^25^

The study findings have important implications for healthcare providers and policymakers. Multiple studies have shown a discrepancy between the number of patients who had positive coeliac serology and those who subsequently underwent endoscopy and biopsies.^30^, ^31^, ^32^, ^33^ Although the lack of referral for endoscopy is multifactorial, patients who refuse or are unable to undergo this invasive procedure often find themselves without alternative diagnostic options. Consequently, many of these patients remain undiagnosed or prematurely start following a gluten‐free diet without the necessary dietetic guidance and medical follow‐up.^33^ Therefore, providing patients with options that align with their preferences to make informed decisions about their care could lead to reduced delays in diagnosis, increased patient satisfaction and better adherence to treatment. Adopting this patient‐centred approach as an optional diagnostic pathway in clinical guidelines could streamline the diagnostic process of coeliac disease for many patients and reduce healthcare utilisation and costs.^34^ Future studies should explore the long‐term outcomes of patients diagnosed based on serology alone and the impact of adopting this non‐invasive strategy on coeliac disease epidemiology.

This novel study has several strengths. First, we used a rigorous methodology to develop and report the DCE according to the current recommendations and guidelines.^15^, ^35^ Second, the attributes and levels included were carefully selected based on the best available evidence, our clinical experience and direct feedback from patients. This approach ensured that the choice sets were evidence‐based, realistic and highly relevant to the preferences and concerns of patients with coeliac disease. Third, the study included a relatively large cohort of patients with suspected or confirmed coeliac disease from various regions across the UK. Finally, we used complex statistical analyses, including mixed logit models and latent class analysis, to account for potential heterogeneity within patient responses and to identify the distinct clinical profiles of patients according to their individual preferences.

Our study had some limitations. As the survey was concluded after exceeding the target number of responses, it is likely that respondents were the most motivated and well‐informed about the topic and scenarios proposed. This could have introduced a selection bias. Respondents were primarily older, white females, which may limit the generalisability of our findings to other ethnic groups or younger populations. However, except for sex, demographics did not significantly influence patient preferences. While the education levels of the respondents were mostly high or very high, these levels are comparable to those of the UK adult population, as per the latest national census data.^36^ The diagnosis of coeliac disease in the current study was self‐reported and not verified against medical records. However, participants were not provided an incentive to take part in the study and were all members of Coeliac UK charity. Additionally, the cross‐sectional nature of the survey limits our ability to observe changes in preferences over time, which could be influenced by several factors. Lastly, despite the robust design of the DCE, the hypothetical choice sets used may not entirely capture the nuances and real‐life complexities of individual diagnostic preferences and decisions. The need for brevity in explaining the choices and the complexity of the matter may have influenced the participants' understanding and responses.

In conclusion, patients prefer the no‐biopsy approach over the biopsy‐based diagnosis of coeliac disease. Diagnostic test accuracy, waiting time to start treatment, and discomfort levels during the procedure were the most important attributes influencing patient preferences. These findings highlight the importance of shared decision‐making and offering patients options that align with their preferences to improve adherence to recommended diagnostic and treatment plans.

## CONFLICT OF INTEREST STATEMENT

MGS and HAP received speaker honoraria from Thermo Fisher.

## ETHICS APPROVAL

The study was reviewed and approved by the South Central—Oxford C Research Ethics Committee (23/SC/0431) and the University of Sheffield Ethics Committee (Reference No. 055734).

## Supporting information

Supporting Information S1

Supporting Information S2

## Data Availability

Data are available upon reasonable request.
