# Supplementary material for: Patient preferences for the diagnosis of coeliac disease: A discrete choice experiment
Source: United European Gastroenterol J. 2024 Aug 27;13(3):330–7. doi: 10.1002/ueg2.12651 (PMC11999034; doi:10.1002/ueg2.12651)
Supplement: Supplementary file 1 — Supporting Information S1 [file UEG2-13-330-s001.docx]

# Supplementary Table 1 – The results of the mixed logit model of patient preferences for the diagnosis of coeliac disease

|  | **Mixed logit model**  **coefficient**  **(Standard error)** | **Standard deviation (standard error)** |
| --- | --- | --- |
| **Serology** | 1.543***  (0.352) | 4.841***  (0.397) |
| **Waiting time to start treatment** | |  |
| 1 month | -1.185***  (0.200) | 0.742*  (0.345) |
| 2 months | -1.321***  (0.203) | -0.096  (0.199) |
| 3 months | -1.145***  (0.280) | 0.891**  (0.310) |
| 6 months | -2.987***  (0.326) | 1.246***  (0.331) |
| **Risk of wrong diagnosis** | |  |
| 5% | -1.659***  (0.191) | 0.380  (0.377) |
| 35% | -6.156***  (0.505) | -3.825***  (0.434) |
| **Risk of missed diagnosis** | |  |
| 2% | -0.957***  (0.179) | 0.522  (0.381) |
| 10% | -2.439***  (0.257) | 1.440***  (0.322) |
| **Perforation risk 0.02%** | 0.118  (0.140) | 0.207  (0.399) |
| **Level of discomfort** | |  |
| Moderate | -0.206*  (0.088) | 0.067  (0.300) |
| High | -1.548***  (0.197) | 1.170***  (0.301) |

* p < 0.05, ** p < 0.01, *** p < 0.001

# Supplementary Table 2 – Willingness to accept the risk of a wrong diagnosis across classes using marginal rates of substitution (MRS)

|  | **Class 1**  **MRS (95% confidence intervals)** | **Class 2**  **MRS (95% confidence intervals)** |
| --- | --- | --- |
| **Serology** | 1.49  (1.31, 1.67) | -1.03  (-1.44, -0.63) |
| **Waiting time to start treatment** | | |
| 1 month | -0.33  (-0.53, -0.13) | -0.59  (-0.90, -0.28) |
| 2 months | -0.33  (-0.54, -0.12) | -0.48  (-0.77, -0.19) |
| 3 months | -0.26  (-0.51,0.002) | -0.13  (-0.52,0.26) |
| 6 months | -0.77  (-0.96, -0.57) | -0.87  (-1.34, -0.40) |
| **Risk of missed diagnosis** | -0.32  (-0.40, -0.23) | -0.55  (-0.70, -0.41) |
| **Perforation risk 0.02%** | 0.05  (-0.06,0.17) | 0.004  (-0.21,0.22) |
| **Discomfort level** | | |
| Moderate | -0.08  (-0.15, -0.01) | -0.07  (-0.20,0.06) |
| High | -0.38  (-0.53, -0.23) | -0.66  (-0.90, -0.41) |
