# Supplementary material for: Patient preferences for the diagnosis of coeliac disease: A discrete choice experiment
Source: United European Gastroenterol J. 2024 Aug 27;13(3):330–7. doi: 10.1002/ueg2.12651 (PMC11999034; doi:10.1002/ueg2.12651)
Supplement: Supplementary file 2 — Supporting Information S2 [file UEG2-13-330-s002.pdf]

Introduction

Hello,

We invite you to take part in a research study that is being conducted by Professor David Sanders and Dr Mohamed Shiha from Sheffield Teaching Hospital NHS Foundation Trust and the University of Sheffield. This survey aims to gain insights into patients' preferences regarding coeliac disease diagnosis. The questionnaire results will inform us whether patients prefer the conventional or no-biopsy pathways for diagnosis. Completing the questionnaire will take approximately 10 minutes. We will not collect any identifiable information about you. The personal information collected will include your age, sex, education level, employment status and geographical location, whether you have coeliac disease, and if you have prior experience with endoscopy. We plan to publish the results in a medical journal and present them at national conferences.

This study has been reviewed and given approval by the Health Research Authority, NHS Research Ethics Committee (23/SC/0431) and The University of Sheffield Ethics Committee (055734).

If you would like further information about this study, please contact Dr Mohamed Shiha. Email: mohamed.shiha1@nhs.net

The decision to take part in this study sits entirely with you, and taking part in this survey is voluntary.

If you submit this survey, it will be understood that you have consented to participate.

Coeliac disease is a common condition that affects 1-in-100 people. The diagnosis of coeliac disease depends on clinical suspicion, blood tests showing raised antibodies directed against gluten and endoscopy with biopsy from the small intestine to prove the damage to the intestinal lining. Endoscopy is an invasive medical procedure whereby a small camera is inserted through the mouth to look inside the stomach and small intestine and take samples. Many people require sedation or anaesthesia and take time off work or make arrangements to accommodate the procedure. Endoscopy is also not without risks, as it carries a small risk of bleeding and causing damage to the gastrointestinal tract.

The antibody tests for coeliac disease are among the most accurate tests for all autoimmune conditions. Recent studies found that when the levels of the antibodies are very high (more than 10 times the upper limit of normal [ULN]), damage to the intestinal lining is almost certain, and endoscopy may not be required in all cases. The questionnaire results will inform us whether patients prefer the conventional pathway for diagnosis in blue or the no-biopsy pathway in green (Figure 1).

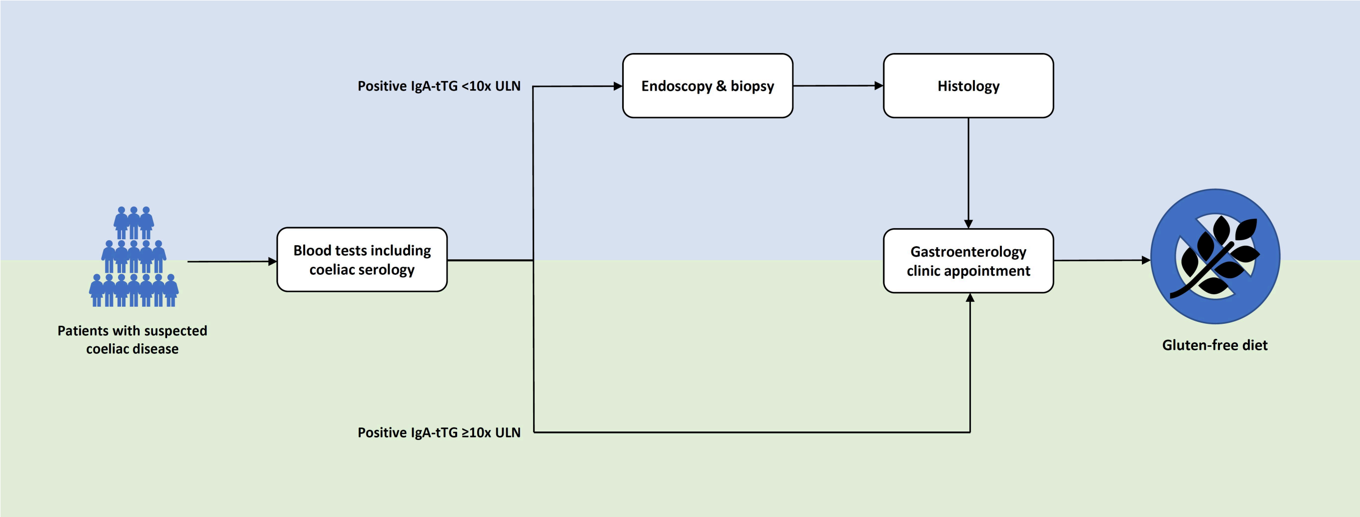

Figure 1: Diagram showing the conventional pathway for diagnosis of coeliac disease in blue or the no-biopsy pathway in green.

The questionnaire contains hypothetical (imaginary) choice sets: each choice contains the same 2 options (Blood test and endoscopy with biopsy).

Each choice set will have different benefits and risks for each option; we would like you to read these different benefits and risks and decide which diagnostic option you prefer.

On the following page, there is an explanation of all the terms used in the questionnaire.

Explanations of all the terms used in the questionnaire

Options	Definitions

**Blood test**	This refers to coeliac antibody tests (IgA tTG) with very high levels (10 times the upper limit of normal).<br>This is a simple blood test that does not require fasting or any preparation.
**Endoscopy with biopsy**	This refers to diagnostic upper gastrointestinal endoscopy with biopsy whereby a small camera is inserted through the mouth to look inside the stomach and small intestine and take samples.<br>The test requires 6-hour fasting and you get to choose whether to have sedation or not.
**Risk of wrong diagnosis**	This is the risk of being told you have coeliac disease when you do not actually have it.<br>Example: The test has 95% accuracy, which means that 1 in 20 patients may get a wrong diagnosis of coeliac disease.
**Risk of missed diagnosis**	This is the risk of being told you do not have coeliac disease when you actually have it.<br>Example: The test results came back normal due to an error, but you actually have coeliac disease.
**Waiting time to start treatment**	This is the time between having the test and starting treatment.<br>Example: It will take six months between you having a blood test and being diagnosed with coeliac disease and starting treatment with a gluten-free diet.
**Risk of bleeding (complication)**	This is the risk of internal bleeding during or after endoscopy. The risk is very small, occurring in approximately 1 in every 1,000 people. This often settles without treatment.
**Risk of perforation (complication)**	This is the risk of endoscopy causing a small tear in the lining of the oesophagus, stomach or small bowel. Nationally, this happens in less than 1 in 5,000 people. This would require a short stay in hospital with antibiotics or may require an operation to repair the tear.
**Discomfort or pain**	This is the risk of discomfort or pain during the blood test or endoscopy. It is common to have discomfort or distress due to the passage of the endoscope and distention (bloating) with air. It is usually short lasting but 1 in every 1,000 patients may be admitted due to ongoing discomfort or pain.

This is a sample question. During the survey, you will be shown 12 different scenarios

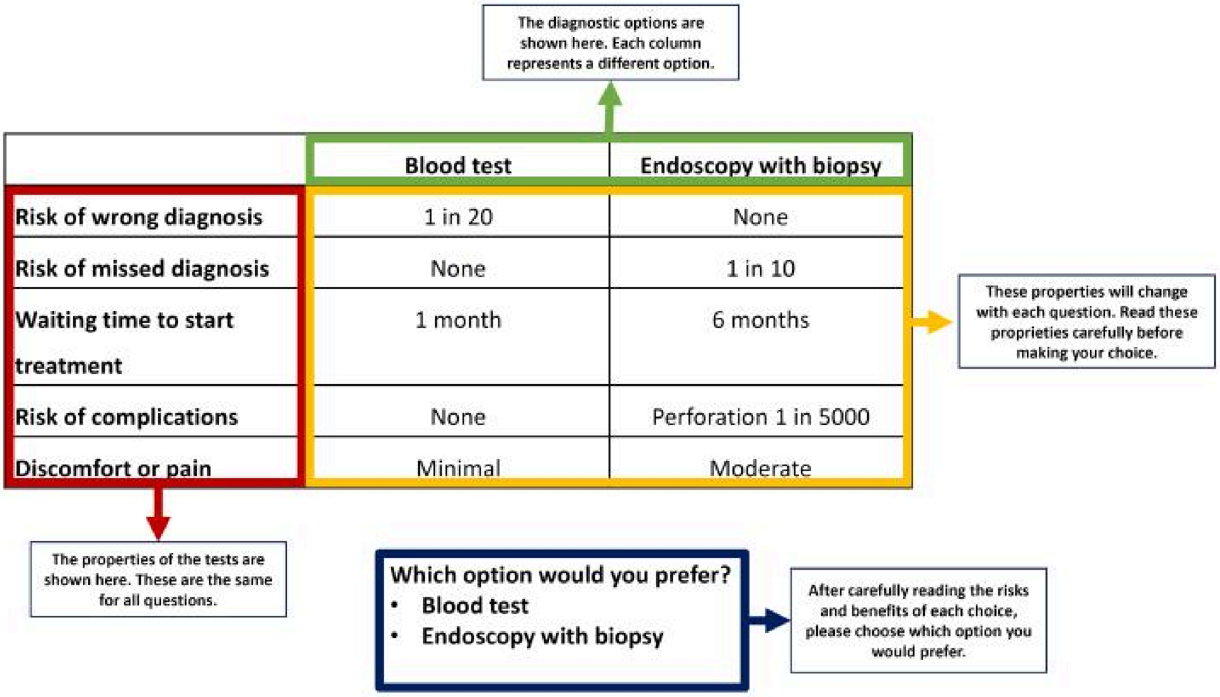

DCE block 1

Please carefully review the options detailed below, then please answer the question.

	**Endoscopy with biopsy**	**Blood test**
**Risk of wrong diagnosis**	None	2 out of every 100 people (2%)
**Risk of missed diagnosis**	10 out of every 100 people (10%)	None
**Waiting time to start treatment**	6 months	1 month
**Risk of complications**	Perforation (0.02%)	None

**Discomfort/pain**	Moderate	Minimal

Which of these choices do you prefer?

- ○ Endoscopy with biopsy
- ○ Blood test

Please carefully review the options detailed below, then please answer the question.

	**Endoscopy with biopsy**	**Blood test**
**Risk of wrong diagnosis**	None	35 out of every 100 people (35%)
**Risk of missed diagnosis**	10 out of every 100 people (10%)	None
**Waiting time to start treatment**	2 months	1 month
**Risk of complications**	Bleeding (0.1%)	None
**Discomfort/pain**	Minimal	Moderate

Which of these choices do you prefer?

- ○ Endoscopy with biopsy
- ○ Blood test

	Endoscopy with biopsy	Blood test
**Risk of wrong diagnosis**	None	5 out of every 100 people (5%)
**Risk of missed diagnosis**	None	None
**Waiting time to start treatment**	3 months	2 months
**Risk of complications**	Perforation (0.02%)	None
**Discomfort/pain**	Minimal	Moderate

Which of these choices do you prefer?

- ◯ Endoscopy with biopsy
- ◯ Blood test

Please carefully review the options detailed below, then please answer the question.

	Endoscopy with biopsy	Blood test
**Risk of wrong diagnosis**	None	5 out of every 100 people (5%)
**Risk of missed diagnosis**	None	None
**Waiting time to start treatment**	2 months	1 month

**Risk of complications**	Bleeding (0.1%)	None
**Discomfort/pain**	High	Minimal

Which of these choices do you prefer?

- ◯ Endoscopy with biopsy
- ◯ Blood test

Demographic questions

How old are you?

- ◯ <25
- ◯ 25 - 34
- ◯ 35 - 44
- ◯ 45 - 55
- ◯ >55

What is your sex?

- ◯ Male
- ◯ Female
- ◯ Prefer not to say

What is your ethnicity?

- ◯ Asian or Asian British
- ◯ Black, Black British, Caribbean or African

- White
- Other ethnic background
- Prefer not to say

What is your education level?

- High school or below
- College or technical school
- University or postgraduate

What is your employment status?

- Student
- Employed, full-time or part time
- Unemployed
- Retired

Where is your current residence in the UK?

- South of England
- Midlands
- North of England
- Scotland
- Wales
- Northern Ireland

Have you been formally diagnosed with coeliac disease?

- ◯ Yes
- ◯ No

Did you ever have an endoscopy?

- ◯ Yes
- ◯ No

Powered by Qualtrics
